# A Self-Temperature Compensation Barometer Based on All-Quartz Resonant Pressure Sensor

**DOI:** 10.3390/s24082460

**Published:** 2024-04-11

**Authors:** Dongxiang Han, Shenfang Yuan, Congwei Feng, Ting Yang

**Affiliations:** 1 Research Center of Structural Health Monitoring and Prognosis, State Key Laboratory of Mechanics and Control for Aerospace Structures, Nanjing University of Aeronautics and Astronautics, Nanjing 210016, China; dxhan_mems@163.com; 2 Beijing Research Institute of Telemetry, Beijing 100076, China; andrewfine@semi.ac.cn; 3 College of Electronic and Information Engineering, Nanjing University of Aeronautics and Astronautics, Nanjing 210016, China; cwfeng@nuaa.edu.cn; 4 School of Integrated Circuits, Tsinghua University, Beijing 100084, China

**Keywords:** barometer, quartz crystal resonator, MEMS, high accuracy, temperature compensation, finite element method (FEM)

## Abstract

This paper reports a self-temperature compensation barometer based on a quartz resonant pressure sensor. A novel sensor chip that contains a double-ended tuning fork (DETF) resonator and a single-ended tuning fork (SETF) resonator is designed and fabricated. The two resonators are designed on the same diaphragm. The DETF resonator works as a pressure sensor. To reduce the influence of the temperature drift, the SETF resonator works as a temperature compensation sensor, which senses the instantaneous temperature of the DETF resonator. The temperature compensation method based on polynomial fitting is studied. The experimental results show that the accuracy is 0.019% F.S. in a pressure range of 200~1200 hPa over a temperature range of −20 °C~+60 °C. The absolute errors of the barometer are within ±23 Pa. To verify its actual performance, a drone flight test was conducted. The test results are consistent with the actual flight trajectory.

## 1. Introduction

Barometers are a key component of the navigation systems of unmanned aerial vehicles (UAVs). By measuring the air pressure value (generally less than 1010 hPa), the flight altitude of UAVs can be calculated [1,2,3]. In order to improve their navigation accuracy, high-precision measurement of the pressure is necessary for sensors. In addition, the ambient temperature also changes significantly with the flight of UAVs. These changes lead to a temperature drift in the sensors’ performance. Therefore, sensors with high precision and excellent temperature stability are needed. A key component of barometers is their absolute pressure sensor. With the development of MEMS technology, more and more types of pressure sensors have been developed. According to the differences in their working principles, pressure sensors can be divided into different types, such as piezoresistive, strain, capacitive, piezoelectric, and resonant sensors [3,4,5,6,7].

The piezoresistive pressure sensor is the main type of sensor used [8,9,10,11]. A Wheatstone bridge was fabricated on a piezoresistive layer. Its fabrication process and signal demodulation are easy. However, it has insufficient long-term stability. The capacitive pressure sensor is another major type of sensor used [12,13,14]. It is easily fabricated and has low power consumption. However, its capacitive structure has high nonlinearity.

Compared to other types, resonant pressure sensors are more attractive for higher accuracy and long-term stability and can achieve 0.05% F.S. These sensors usually employ resonators, such as tuning forks, as resonant strain gauges to sense pressure-induced stresses in the diaphragm. The output signal is a digital-type frequency. Among the resonant pressure sensors, quartz crystal resonant pressure sensors are the most popular [15,16,17,18].

Frequency drift with the temperature is a main limitation of sensor performance. The main parameters of a quartz structure, such as its Young’s modulus, density, and size, vary with the temperature. This leads to a significant temperature drift and accuracy decrease. In order to improve the accuracy over a temperature range, compensation techniques, such as temperature sensors, the quality factor (Q-factor), and differential output, have been adopted. However, these methods have their own drawbacks in the compensation processes. Wang, J. et al. reported a resonant pressure sensor that employs a temperature sensor for temperature compensation [19,20]. This method is only suitable for a slowly varying temperature. For a rapid varying temperature, there is a serious time delay, which leads to reduced accuracy. Hopcroft et al. reported another compensation method using the Q-factor as a thermometer [21]. The Q-factor of resonators is a function of temperature and an ideal temperature-sensing method. However, this method has strict requirements for the Q values and is not effective for sensors with a lower Q. Zhang, Q. et al. reported a differential output method [22]. Two consistent resonators are needed to provide differential output. However, it is difficult to fabricate two resonators of identical size.

To realize temperature compensation and reduce the difficulty of fabrication, a novel design of a quartz sensor is proposed in this paper. A DETF and a SETF are designed. Additionally, a self-temperature compensation approach based on the two frequencies is developed to improve the performance of the barometer.

## 2. Sensor Design and Simulation

**Figure 1:**
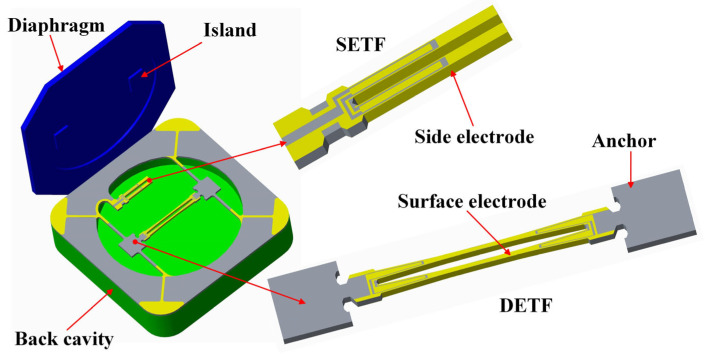
Schematic image of the sensing unit.

In response to the usage requirements of drone barometers, an absolute pressure sensor with a range of 1200 hPa was designed. This resonant pressure sensor is an all-quartz structure. It contains three parts: a quartz crystal diaphragm, quartz crystal resonators, and a quartz crystal back cavity, as shown in Figure 1. The DETF and SETF were designed for the quartz crystal resonator parts. The DETF works as the pressure sensor, and the SETF works as the temperature compensation sensor. The diaphragm deforms in response to applied pressure. Two islands were designed to transfer and increase the pressure to the DETF. The resonance frequency of the DETF is positively proportional to the pressure. Meanwhile, the SETF was designed as single-ended to effectively avoid the influence of pressure. When the temperature changes, the resonance frequency of the DETF and SETF will show a negative correlation to the temperature. The back cavity structure includes a trench that provides room for the deformation of the resonators.

**Figure 2:**
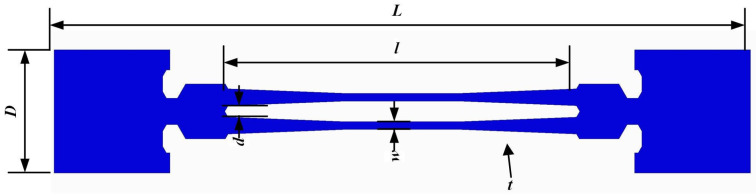
Parameters of the DETF quartz resonator.

The DETF was designed and analyzed in our previous work in detail. This article provides a brief description in this section. To avoid low-frequency interference (≤20 kHz), the resonance frequency of the DETF (*f_D_*) was designed as 45 kHz. The detailed parameters are shown in Figure 2 and Table 1.

**Figure 3:**
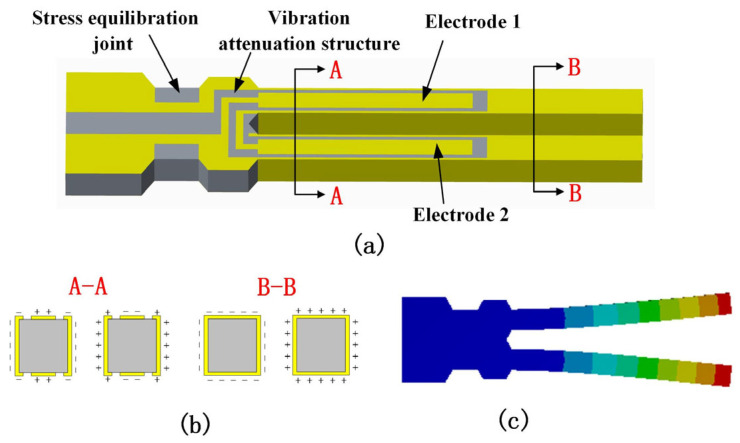
(**a**) Schematic of the quartz crystal SETF resonator; (**b**) electric field in the cross section; (**c**) finite element analysis of the SETF resonator.

Figure 3 shows the schematic of the SETF. Finite element analysis was used to simulate the resonant mode and optimize the structure size and electrodes. The SETF length and width are along the y and x directions of the Z-cut quartz crystal wafer, respectively. The electrodes are arranged as shown in Figure 3a, and they can obtain the in-plane vibration mode. The vibration attenuation structure and stress equilibration joint are designed to reduce the energy loss from the SETF to the diaphragm. This design is beneficial for a high Q value of the SETF.

**Figure 4:**
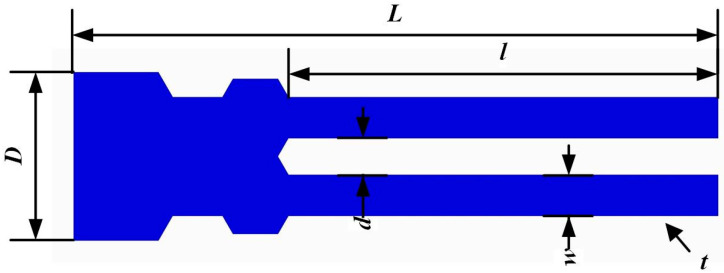
Parameters of the SETF quartz resonator.

To avoid frequency interference with the DETF, the resonance frequency of the SETF was designed as 30 kHz. The detailed parameters of the SETF are presented in Figure 4 and Table 2. The SETF was designed and calculated by FEM.

**Figure 5:**
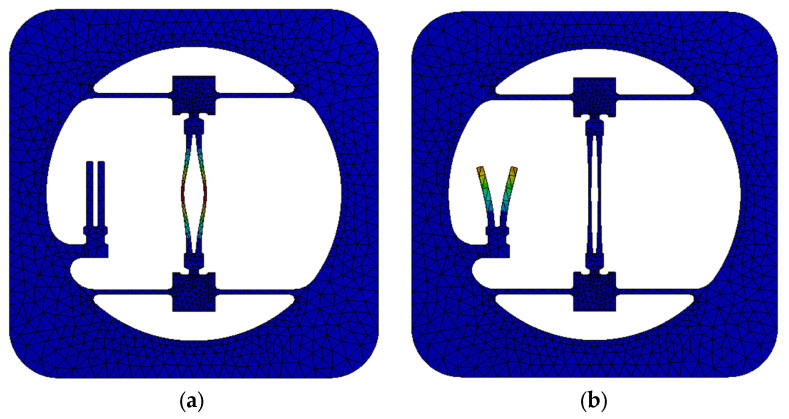
The resonant modes of DETF (**a**) and SETF (**b**).

The resonant modes of the DETF and SETF are as shown in Figure 5. The in-plane antiphase vibration of the resonators reduces the energy loss effectively. As the difference between the two resonance frequencies is significant, the two resonant modes do not interfere with each other.

**Figure 6:**
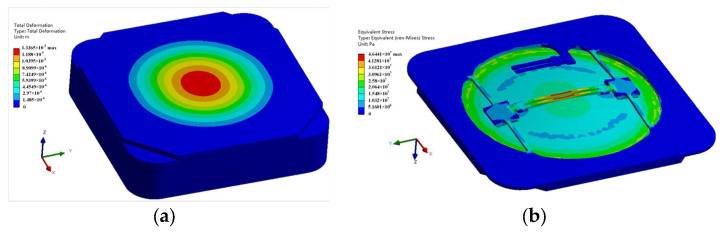
(**a**) Deformation of the resonant pressure sensor under 1200 hPa; (**b**) stress distribution of the resonant pressure sensor under 1200 hPa.

The deformation of this pressure sensor when a pressure of 1200 hPa is applied to the diaphragm is shown in Figure 6a. The thickness of the diaphragm is 400 μm. The maximum displacement is 13.36 μm, which is far less than 1/10 of the diaphragm thickness. This indicates that the design can meet the linear elastic theory. The stress distribution is shown in Figure 6b, with a maximum stress of 46.44 MPa, which is lower than the allowable stress of quartz. The maximum stress is distributed in the center of the DETF, which is beneficial for high sensitivity. Meanwhile, there is no stress in the SETF. This ensures that the resonance frequency of the SETF (*f_S_*) is not affected by pressure. The *f_S_* is only related to the temperature.

## 3. Self-Temperature Compensation

Generally, the resonance frequency of resonators is affected by the pressure and temperature and can be expressed by the function *f = f*(*P*,*T*)** [23]. In order to improve the accuracy of the full temperature and decrease the difficulty of temperature compensation, the DETF and SETF are designed on the same diaphragm. The DETF (Equation (1)) is double-ended and used to detect the pressure. It is affected by the pressure and temperature. The resonance frequency is *f_D_* = *f*(*P*,*T*)**. The SETF (Equation (2)) is single-ended and is only affected by the temperature. It is designed to realize the temperature compensation. The resonance frequency of the SETF is *f_S_ *=* f*(*T*)**.

(1) $$f_{p}=f_{D}=f\left(P,T\right)$$

(2) $$f_{T}=f_{S}=f\left(T\right)$$

In order to achieve temperature compensation, the polynomial fitting method was adopted. The pressure and temperature are expressed by a fourth-order polynomial. Equation (3) shows that the pressure can be simply calculated by *f_p_* and *f_T_*.

(3) $$\begin{array}{l} P_{i}=A\times Y_{i}^{0}+B\times Y_{i}^{1}+C\times Y_{i}^{2}+D\times Y_{i}^{3}+E\times Y_{i}^{4}\;\\ Y_{i}=f_{p_{i}}-f_{p}^{\prime } \end{array}$$

where *P* is the pressure, *T* is the temperature of the sensing unit, *f_p_* is the resonant frequency of the DETF, and *f_T_* is the resonant frequency of the SETF. *i* = 1, 2, 3, 4, 5, and 6. The calibration pressure value is P_i_ = 200 hPa, 400 hPa, 600 hPa, 800 hPa, 1000 hPa, and 1200 hPa. *j* = 1, 2, 3, 4, 5. T_j_ = −20 °C, 0 °C, 20 °C, 40 °C, 60 °C. A_k_, B_k_, C_k_, D_k_, E_k_ (k = 0, 1, 2, 3, 4) *f_T_*′, and *f_p_*′ are the coefficients, as shown in Equation (4).

(4) $$\begin{array}{l} {A=A}_{0}\;\times \;X_{j}^{0}{\;+\;A}_{1}\;\times \;X_{j}^{1}{\;+\;A}_{2}\;\times \;X_{j}^{2}{\;+\;A}_{3}\;\times \;X_{j}^{3}{\;+\;A}_{4}\;\times \;X_{j}^{4}\\ {B=B}_{0}\;\times \;X_{j}^{0}{\;+\;B}_{1}\;\times \;X_{j}^{1}{\;+\;B}_{2}\;\times \;X_{j}^{2}{\;+\;B}_{3}\;\times \;X_{j}^{3}{\;+\;B}_{4}\;\times \;X_{j}^{4}\\ {C=C}_{0}\;\times \;X_{j}^{0}{\;+\;C}_{1}\;\times \;X_{j}^{1}{\;+\;C}_{2}\;\times \;X_{j}^{2}{\;+\;C}_{3}\;\times \;X_{j}^{3}{\;+\;C}_{4}\;\times \;X_{j}^{4}\\ {D=D}_{0}\;\times \;X_{j}^{0}{\;+\;D}_{1}\;\times \;X_{j}^{1}{\;+\;D}_{2}\;\times \;X_{j}^{2}{\;+\;D}_{3}\;\times \;X_{j}^{3}{\;+\;D}_{4}\;\times \;X_{j}^{4}\\ {E=E}_{0}\;\times \;X_{j}^{0}{\;+\;E}_{1}\;\times \;X_{j}^{1}{\;+\;E}_{2}\;\times \;X_{j}^{2}{\;+\;E}_{3}\;\times \;X_{j}^{3}{\;+\;E}_{4}\;\times \;X_{j}^{4}\\ X_{j}=f_{T_{j}}-f_{T}^{\prime } \end{array}$$

The temperature can be simply calculated by *f_T_*, which can be approximated by polynomial fitting, as shown Equation (5). F_k_ (k = 0, 1, 2, 3, 4) are the coefficients.

(5) $$T_{j\;}{=F}_{0}\times f_{T_{j}}^{0}{+F}_{1}\times f_{T_{j}}^{1}{+F}_{2}\times f_{T_{j}}^{2}{+F}_{3}\times f_{T_{j}}^{3}{+F}_{4}\times f_{T_{j}}^{4}$$

## 4. Fabrication

**Figure 7:**
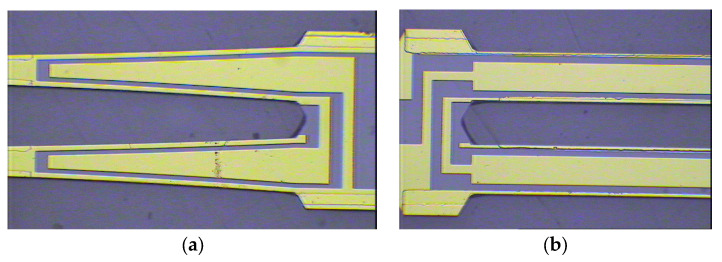
The fabricated DETF (**a**) and SETF (**b**).

The sensor chip was fabricated by quartz MEMS processes. Quartz crystal anisotropic chemical etching and 3D electrode deposition were the main processes used. To fabricate the side electrodes, a shadow mask was used. Cr/Au was deposited and patterned to form the electrodes. The detailed fabrication processes were reported in our previous work. Figure 7 shows the fabricated DETF and SETF.

**Figure 8:**
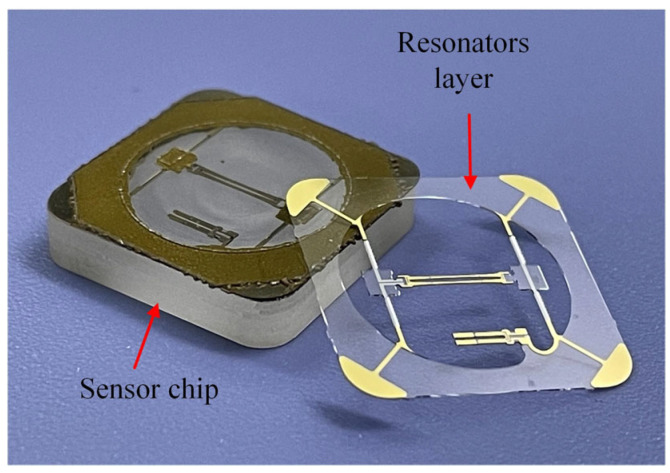
Image of the fabricated sensor chip.

In order to achieve an absolute pressure chamber structure, the glass frit vacuum bonding process was used. The three layers of the quartz crystal diaphragm, quartz crystal resonators, and quartz crystal back cavity were bonded together successively by glass frit. The glass frit bonding between the islands and the DETF resonator obtained a lower hysteresis error and higher stability compared to epoxy resin adhesive. This is because the glass frit and the quartz had a similar thermal expansion coefficient. The thick back cavity structure was used to attenuate the effect of mounting stress on the DETF resonator. The fabricated sensor chip is shown in Figure 8.

**Figure 9:**
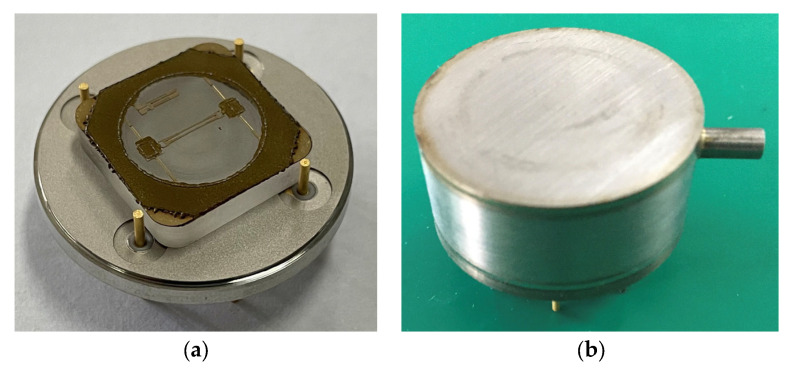
The metallic base (**a**) and packaged pressure sensor (**b**).

Subsequently, the sensor chip was attached to the metallic base with silica gel. A wire bonding process was implemented to connect the pads on the chip to the pins embedded in the metallic base. A stainless steel package was welded to the metallic base. Figure 9 shows the packaged pressure sensor.

**Figure 10:**
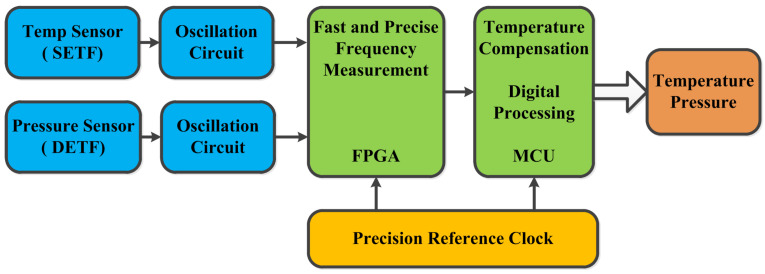
Circuit block diagram.

In order to achieve signal demodulation and temperature compensation, a circuit was designed. Figure 10 shows the functional block diagram. The circuit consists of two oscillators, a precision reference clock, an FPGA, and an MCU. The resonant pressure sensor will output two frequency signals: *f_p_* and *f_T_*. The oscillation circuits can provide stimulus to the resonators and convert the resonance frequency into electrical signals. The precision reference clock provides the FPGA and the MCU with a stable time base. The FPGA is embedded using a digital Σ-Δ technique for measuring the frequency. The MCU provides the functions of temperature compensation and digital processing. The coefficients (A_k_, B_k_, C_k_, D_k_, Ek, and F_k_) were calculated by a calibration test (P_i_ and T_j_) and written into the MCU. The pressure (P), which is temperature-compensated, is a function of *f_p_* and *f_T_*. The temperature (T) is a function of *f_T_*.

**Figure 11:**
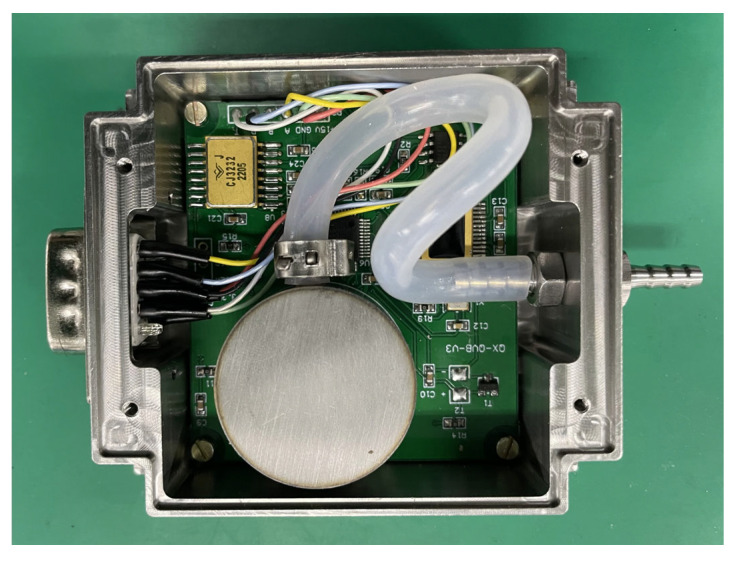
Image of the fabricated barometer.

A barometer was formed by assembling the pressure sensor, circuit, and package together. Figure 11 shows the fabricated barometer.

## 5. Results and Discussion

**Figure 12:**
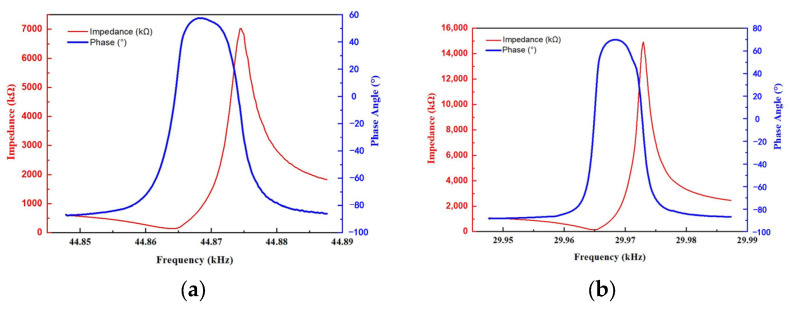
The impedance frequency and phase frequency curves of the quartz resonators. (**a**) DETF resonator; (**b**) SETF resonator.

The resonant pressure sensor was characterized by the Impedance Analyzer (Agilent 42924A, Keysight, Kimballton, IA, USA). The main parameters of the sensor, such as the resonance frequency and quality factor (Q-factor), were achieved by tests and calculations. Table 3 lists the main parameters of the DETF and SETF resonators. This sensor was tested under atmosphere at 25 °C. The resonance frequencies of the DETF and SETF were 44.864 kHz and 29.964 kHz, respectively. Figure 12 shows the impedance frequency and phase frequency curves of the DETF and SETF.

**Figure 13:**
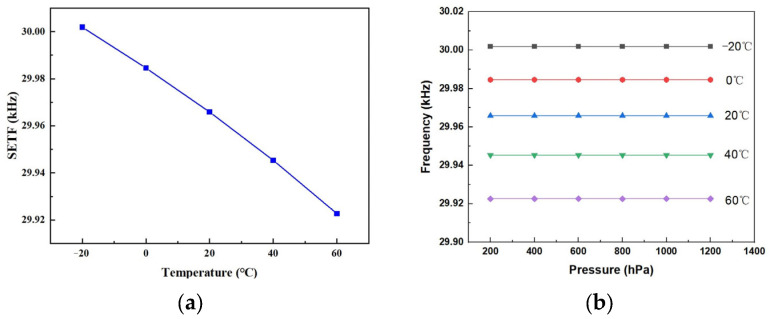
(**a**) The output curves of the SETF resonator vs. temperature; (**b**) the resonance frequency vs. pressure under different temperatures.

The barometer was calibrated by a high-precision calibrator (Druck Pace 6000, GE, Billerica, MA, USA). The pressure sensor and circuits were sealed in a metallic shell chamber. A pipe was used to connect the calibrator to the pressure sensor. To characterize the temperature properties, the barometer was put into a thermostat (Giant Force ECT-408, Giant Force, Taipei, China) to obtain different temperature environments. The barometer was calibrated with a temperature range from −20 °C to +60 °C, with an interval of 20 °C, and a pressure range from 200 hPa to 1200 hPa, with an interval of 200 hPa. Figure 13a shows the output frequency of the SETF with the temperature under atmosphere. The frequency variation was 79.27 Hz. The sensitivity was 0.99 Hz/°C. Figure 13b shows the resonance frequency of the SETF versus the pressure and temperature. The test results indicate that the resonance frequency of the SETF was irrelevant to the pressure, which is consistent with the theoretical design.

**Figure 14:**
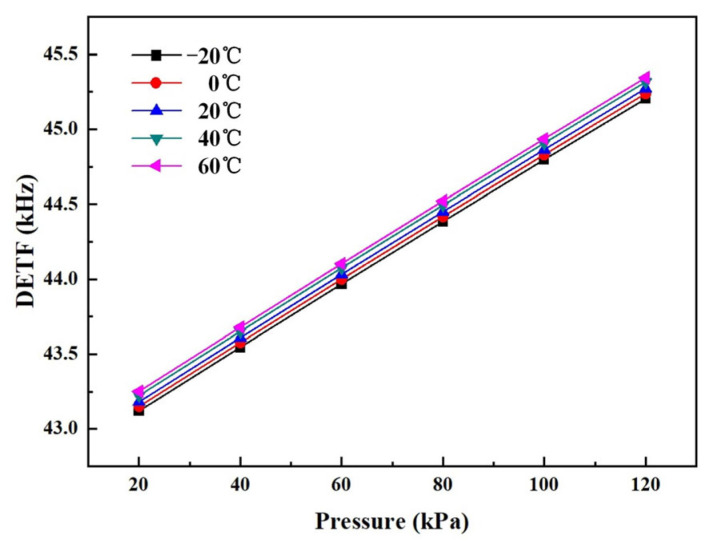
The output curves of the DETF on the full temperature scale before compensation.

Figure 14 shows the output curves of the DETF on the full temperature scale before compensation. The thermal zero shift was 1.65 Hz/°C, and thermal sensitivity shift was 0.01 Hz/hPa/°C. The accuracy of the full temperature range was 6.77%.

**Figure 15:**
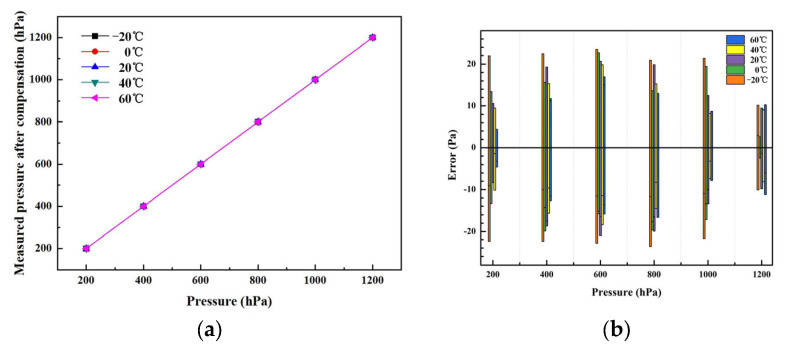
(**a**) The output curves at different temperatures; (**b**) absolute error values for full temperature and pressure ranges.

The coefficients of the polynomial (A_k_, B_k_, C_k_, D_k_, E_k_, F_k_ (k = 0, 1, 2, 3,4)) can be calculated by pressure calibration. Table 4 shows the coefficients as calculated by Origin software. The nonlinear surface fit function of Origin was used. Based on the proposed self-temperature compensation, the accuracy of the barometer reached 0.019% F.S. for the full temperature range. Figure 15a shows the output curves for the full temperature range after compensation. It can be observed that the absolute errors of the developed barometer were within ±23 Pa, as shown in Figure 15b.

**Figure 16:**
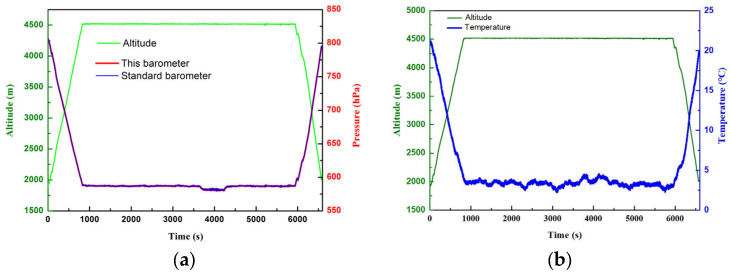
Drone track test data. (**a**) The relationship between the track altitude and pressure. (**b**) The relationship between the track altitude and temperature.

To further verify its feasibility, the barometer was installed on a drone for a track measurement. The flight time of the drone was 6596 s. The altitude increased from 1900 m to 4525 m. The pressure decreased from 810 hPa to 579 hPa, as tested by a standard barometer and the newly designed one. And the temperature decreased from 22 °C to 2 °C, as tested by the SETF. The results are shown in Figure 16. The measurement deviation between the standard barometer and the current one is less than 10 Pa.

## 6. Conclusions

This paper reports a novel barometer based on a quartz crystal resonant pressure sensor. DETF and SETF resonators are designed on the same diaphragm, which work as a pressure sensor and temperature compensation sensor, respectively. Based on the quartz MEMS process, a resonant pressure sensor was fabricated. The pressure range is 200~1200 hPa. A self-temperature compensation method was studied and used in the pressure sensor. After calibration, the accuracy of this sensor was 0.019% F.S. in a temperature range of −20 °C~+60 °C. The barometer was installed on a drone for a track measurement. The test results show that the measurement value is consistent with the theoretical one.

## Figures and Tables

**Table 1:** Dimensions of structural parameters of quartz DETF resonator.

Symbols	Designation	Values
*t*	Thickness of DETF	200 μm
*l*	Length	4800 μm
*w*	Width	96 μm
*d*	Tine-spacing	150 μm
*L*	Overall length	9.6 mm
*D*	Overall width	1.7 mm
*f_D_*	Natural frequency	45 kHz

**Table 2:** Dimensions of structural parameters of quartz SETF resonator.

Symbols	Designation	Values
*t*	Thickness of SETF	200 μm
*l*	Length	2600 μm
*w*	Width	250 μm
*d*	Tine-spacing	220 μm
*L*	Overall length	3.9 mm
*D*	Overall width	1 mm
*f_S_*	Natural frequency	30 kHz

**Table 3:** Main parameters of the pressure sensor under atmosphere at 25 °C.

	DETF	SETF
f (kHz)	44.864	29.964
R_1_ (kΩ)	144.5	139.5
C_1_ (fF)	1.7	1.8
L_1_ (kH)	7.3	15.5
C_0_ (pF)	2.4	2.4
Q	14,215	20,970

**Table 4:** Coefficients of the polynomial after compensation.

A_0_: 6.964961 × 10^2^	A_1_: 9.515319 × 10^−1^	A_2_: −5.538909 × 10^−3^	A_3_: −6.086999 × 10^−5^	A_4_: 4.114796 × 10^−6^
B_0_: 4.778090 × 10^−1^	B_1_: 5.076529 × 10^−5^	B_2_: 8.540680 × 10^−8^	B_3_: −6.179048 × 10^−10^	B_4_: 1.852976 × 10^−10^
C_0_: 6.644117 × 10^−6^	C_1_: −2.461253 × 10^−9^	C_2_: −9.652854 × 10^−11^	C_3_: −8.904153 × 10^−14^	C_4_: 6.339908 × 10^−15^
D_0_: 4.474582 × 10^−10^	D_1_: 5.730680 × 10^−12^	D_2_: 2.478367 × 10^−13^	D_3_: −2.354583 × 10^−15^	D_4_: −1.262200 × 10^−16^
E_0_: 4.542116 × 10^−15^	E_1_: 3.192787 × 10^−15^	E_2_: 5.955707 × 10^−17^	E_3_: −7.483828 × 10^−19^	E_4_: −7.897508 × 10^−21^
F_0_: 8.136063 × 10^−3^	F_1_: −2.202319 × 10^−7^	F_2_: −2.199550 × 10^−3^	F_3_: 1.479485 × 10^−7^	F_4_: −2.487695 × 10^−12^
*f_T_*′**: 29,964.049	*f_p_*′**: 44,238.170			

## Data Availability

Data are contained within the article.
